# Supplementary material for: Structure of ABCB1/P-Glycoprotein in the Presence of the CFTR Potentiator Ivacaftor
Source: Membranes (Basel). 2021 Nov 25;11(12):923. doi: 10.3390/membranes11120923 (PMC8703531; doi:10.3390/membranes11120923)
Supplement: Supplementary file 1 [file membranes-11-00923-s001.zip › membranes-1463127-supplementary.pdf]

membranes

MDPI

Supplementary Material: Structure of ABCB1/P-Glycoprotein in the Presence of the CFTR Potentiator Ivacaftor

Alessandro Barbieri [1,2], Nopnithi Thonghin [1,3], Talha Shafi [1], Stephen M. Prince [1], Richard F. Collins [1] and Robert C. Ford [1,*]

1 School of Biological Sciences, Faculty of Biology Medicine and Health, The University of Manchester, Oxford Road, Manchester M13 9PT, UK; alessandro.barbieri@postgrad.manchester.ac.uk (A.B.); nopnithi@g.swu.ac.th (N.T.); talha.shafi@manchester.ac.uk (T.S.); steve.prince@manchester.ac.uk (S.M.P.); Richard.Collins@manchester.ac.uk (R.F.C.)

2 Bioinformatics Institute (BII), Agency for Science, Technology, and Research (A*STAR), 30 Biopolis Street, #07-01 Matrix, Singapore 138671, Singapore

3 Department of Biology, Faculty of Science, Srinakharinwirot University, 114 Sukhumvit 23, Wattana District, Bangkok 10110, Thailand

* Correspondence: robert.ford@manchester.ac.uk

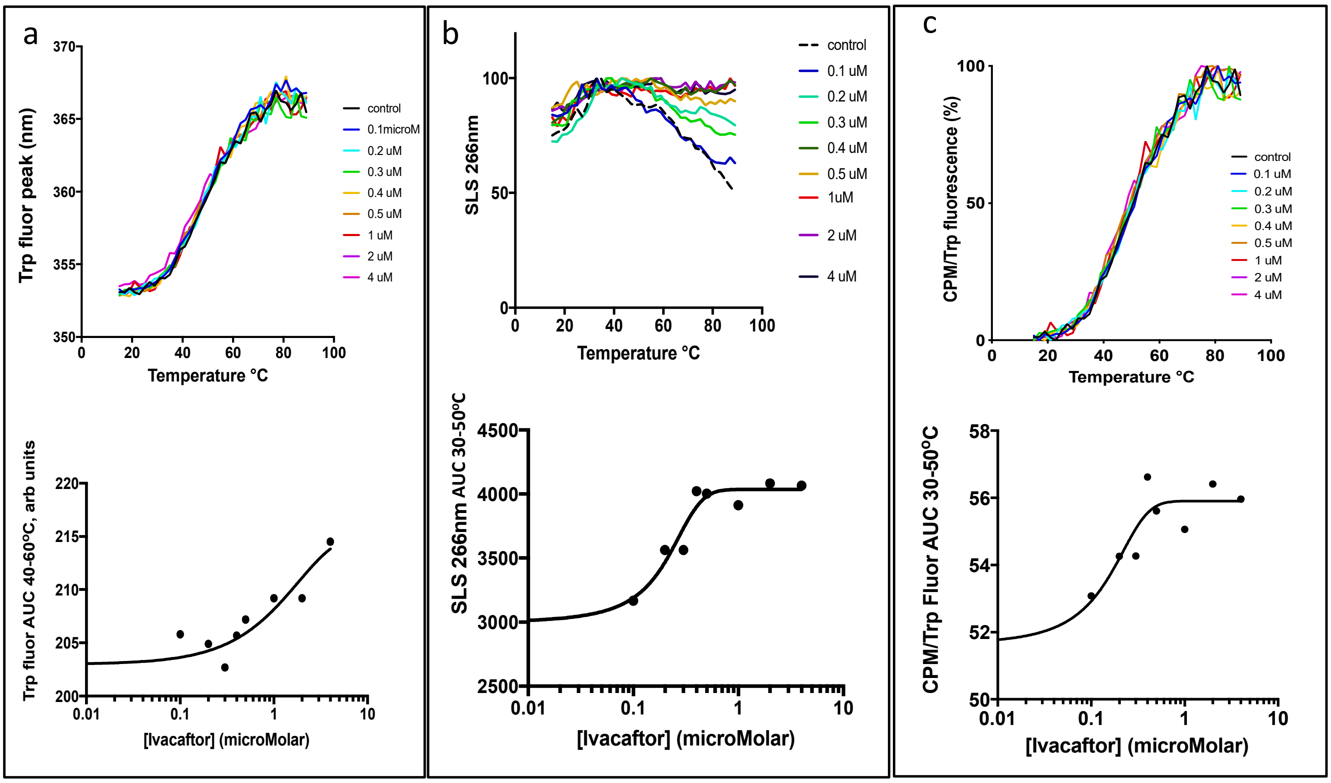

Figure S1. Murine P-gp thermostability and effects of ivacaftor: (a) Ivacaftor binding detected using the intrinsic tryptophan fluorescence peak wavelength of P-gp. A broad protein unfolding transition between 30-50°C was detected and ivacaftor binding exerted a small detectable effect on the area under the curve (AUC) in this range (lower graph). (b) Static light scattering (SLS), measured simultaneously to the tryptophan fluorescence showed a shift from increasing to decreasing light scattering, with an inflexion point around 30-50°C that was sensitive to ivacaftor. Arbitrary units are displayed for SLS. (c) CPM binding to P-gp cysteine residues detected an unfolding transition between 30-50°C that was sensitive to ivacaftor. CPM fluorescence was divided by the tryptophan fluorescence to correct for thermal quenching effects and then normalised. Arbitrary units are displayed for AUC between 30-50 °C.

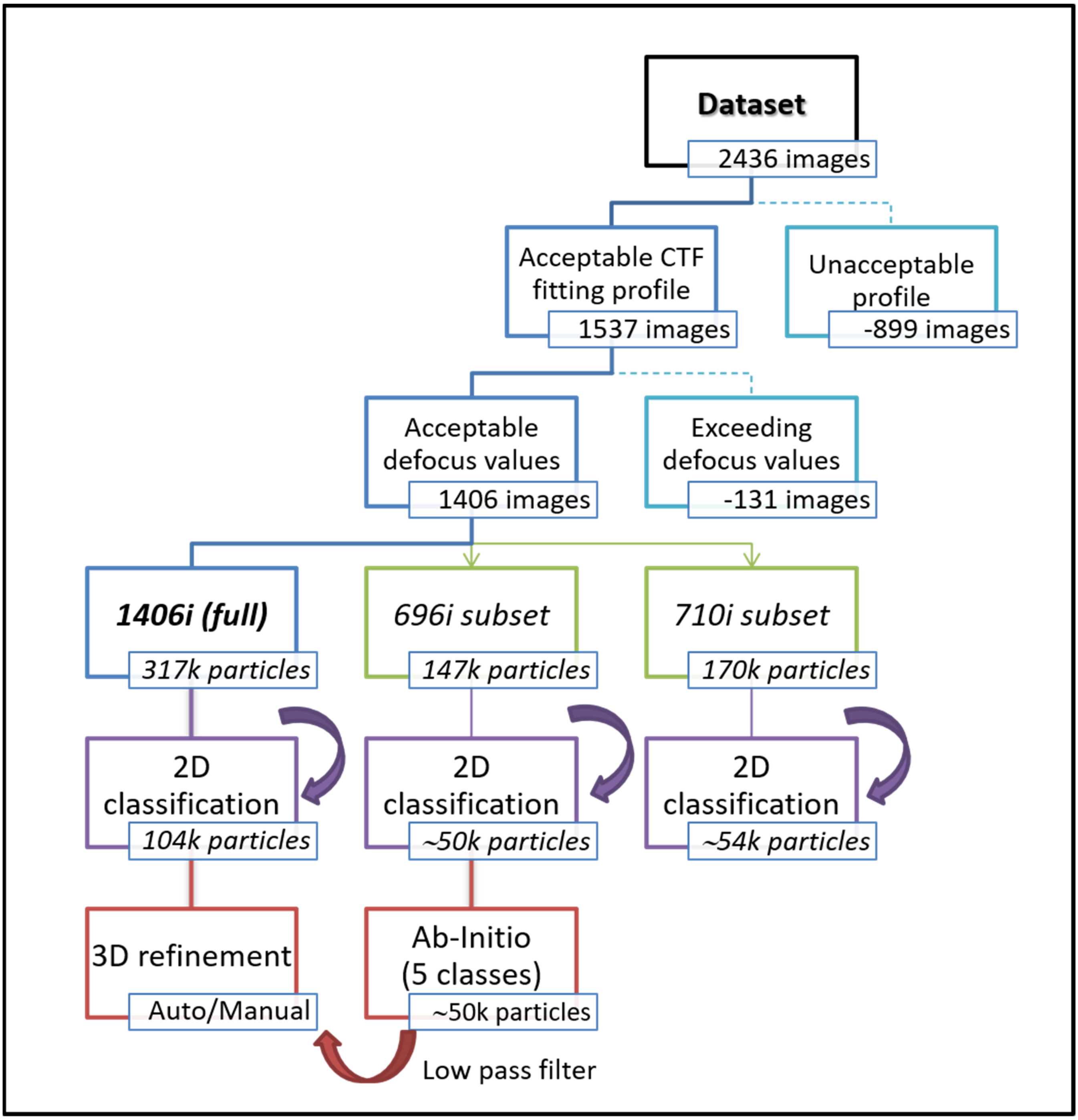

Figure S2. Workflow: From the 2436 images 899 micrographs were removed (with CTF fitting precision to only 6 Ångstrom resolution or worse). A further subset of images, with CTF defocus values indicating astigmatic behavior, were also removed, yielding a final set of 1406 images. These were further divided into two roughly equal subsets to test for the reproducibility of the 2D classification procedure (green boxes). One of these subsets was also employed for generating the five *ab-initio* 3D classes.

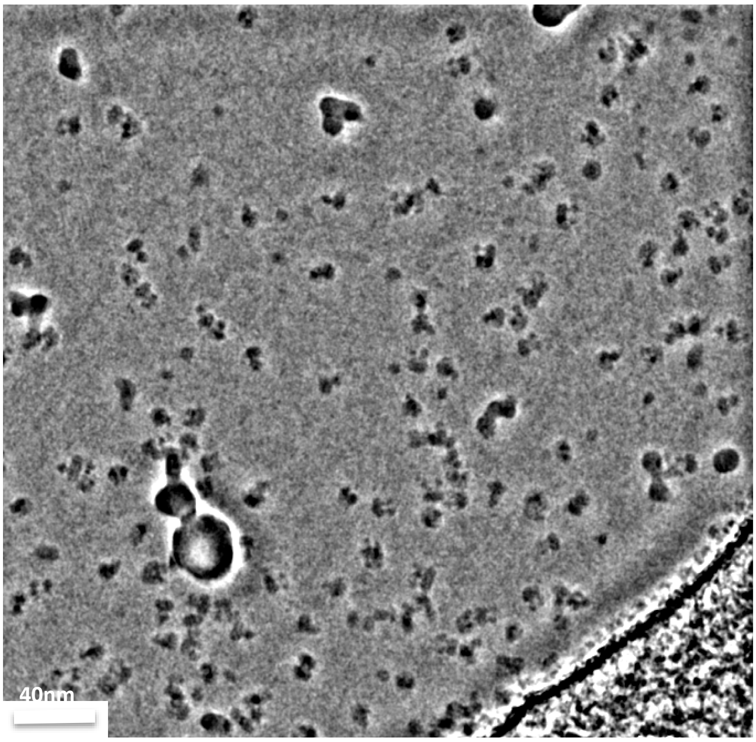

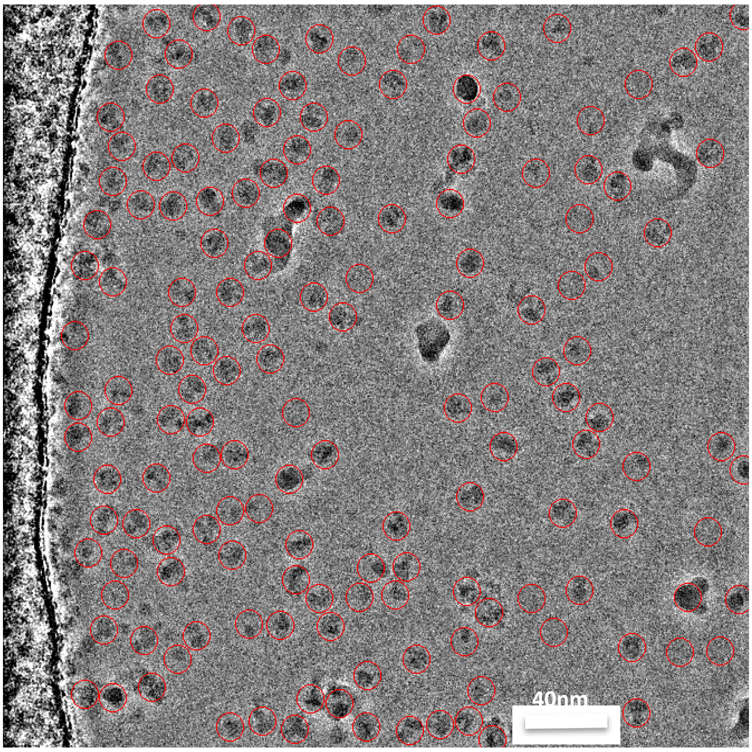

Figure S3. Cryo-EM images initially visualized with the CisTEM software suite. Top: Very high contrast particles can be readily observed due to the use of the Volta phase plate. The defocus used to record the image was ~590nm. Particles were picked and later padded into a final box size of 200 pixels as shown on the lower micrograph. The defocus of the exemplar image (right) was ~780nm.

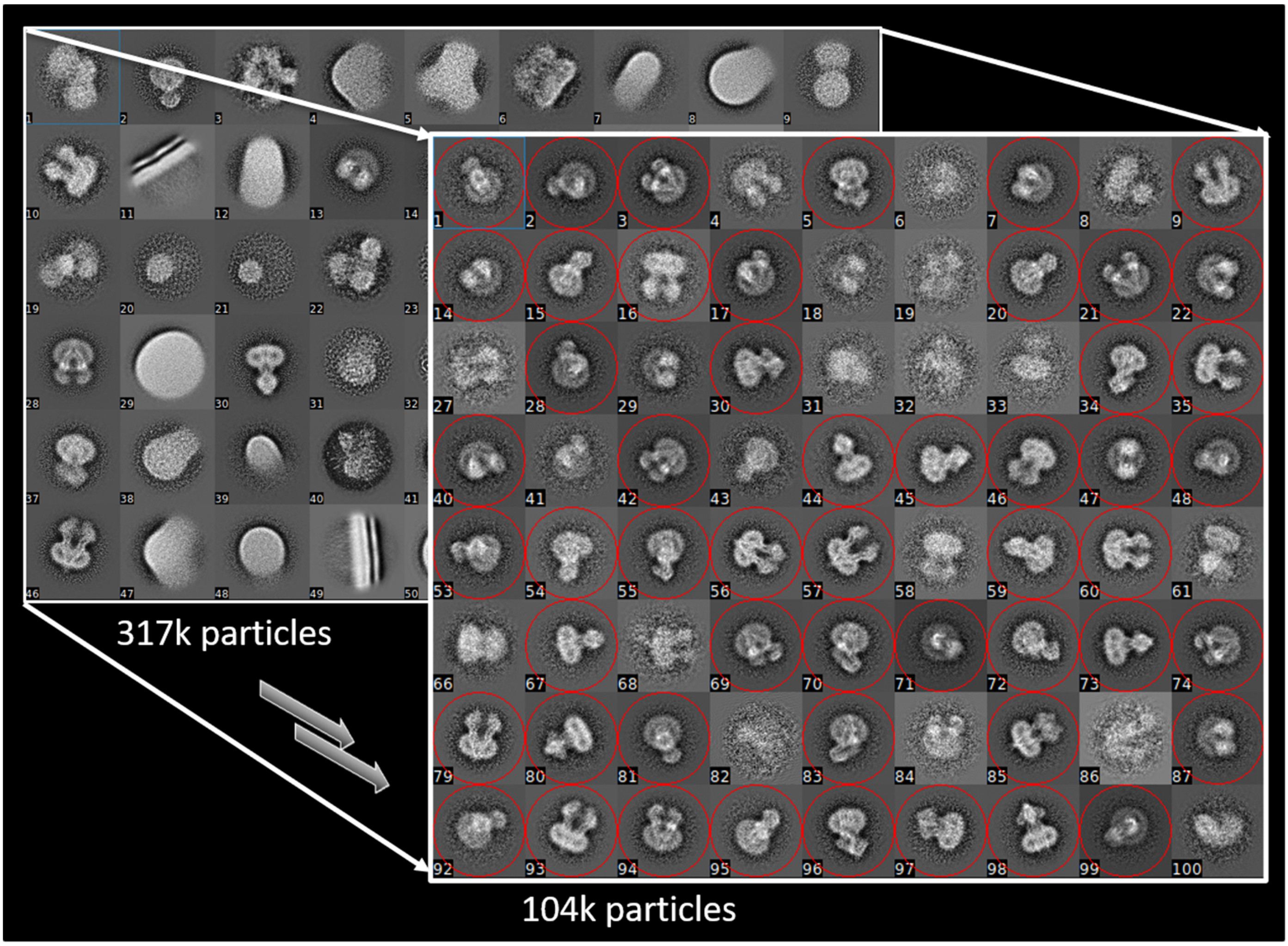

Figure S4. Initial particle classification: (Left) A preliminary reference-free classification of particle projections was carried out and 13 out of 60 classes, corresponding to 108,000 particles, were selected. These were subsequently re-classified allowing up to 100 classes (foreground, right). Particles in classes with poorly defined features were removed, leaving a final dataset of 104,000 particles.

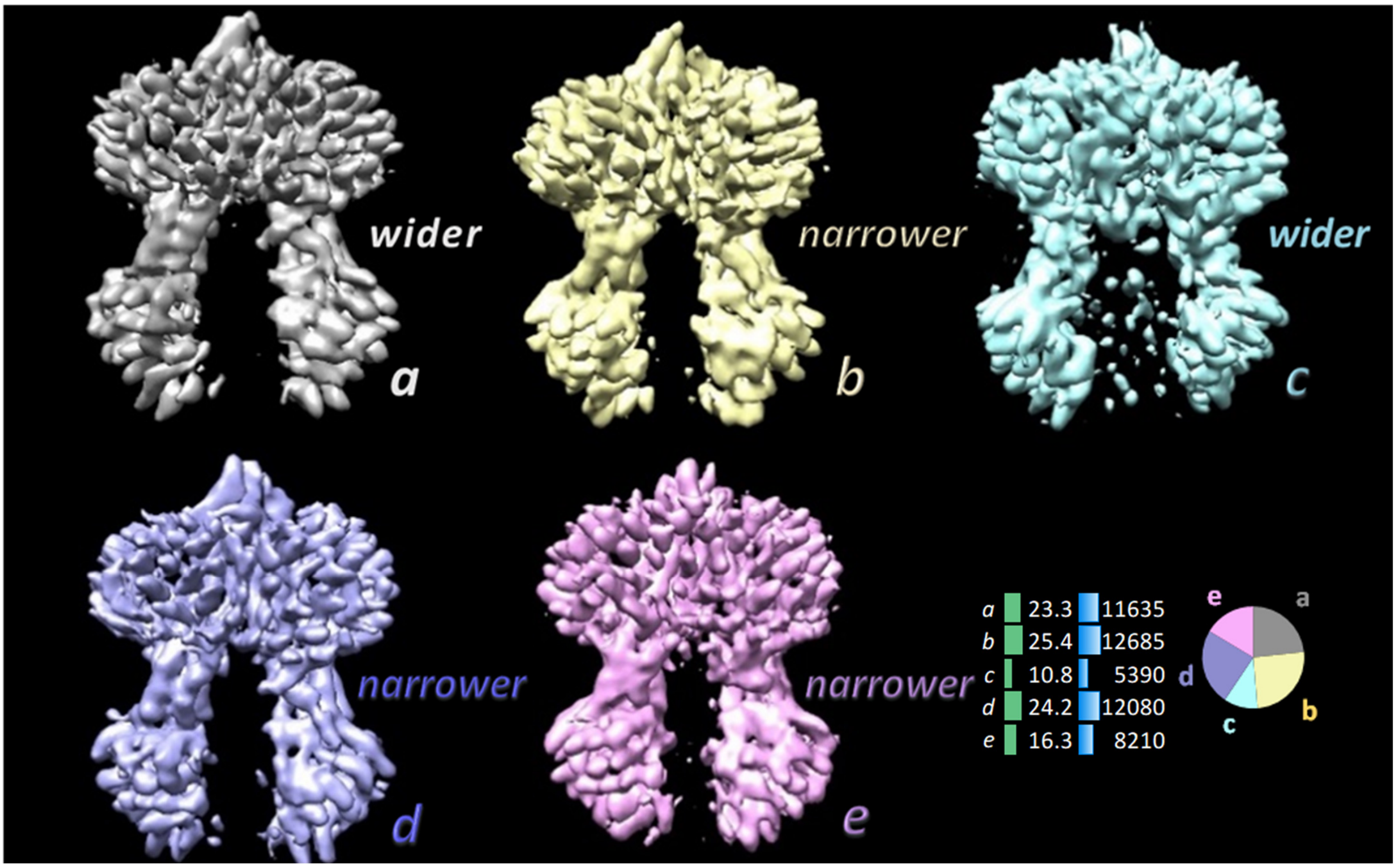

Figure S5. 3D classification of particles. *Ab-initio* 3D classification of a subset of the ABCB1 dataset, which superficially separated the particles into classes with wider or narrower separation of the NBDs. Up to five classes were allowed. At this early stage of the procedure the 3D maps are relatively noisy (e.g., class *c*). Bottom right shows the number of particles for each *ab-initio* class (blue), their percentages (green) and their distribution as a pie chart.

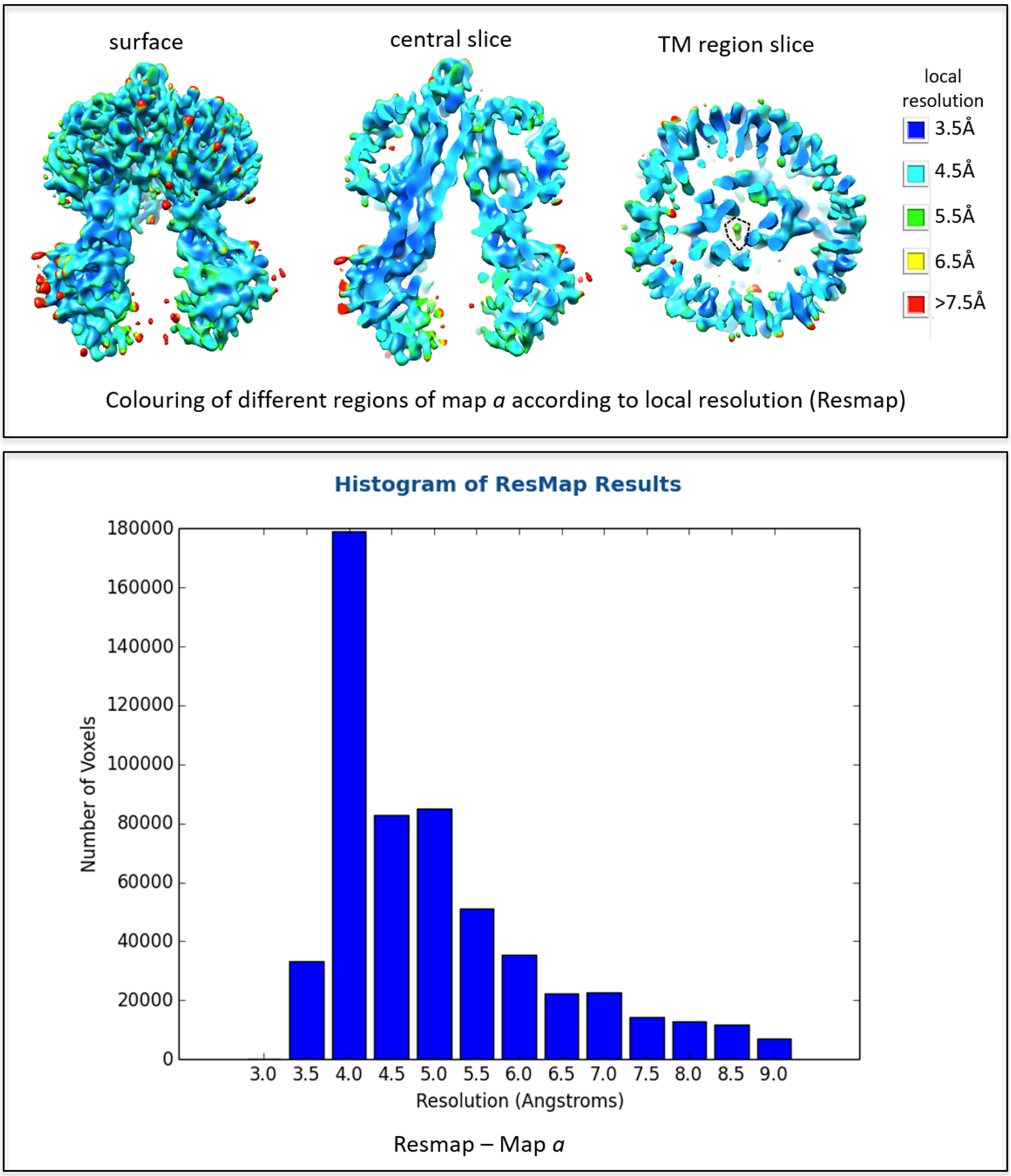

Figure S6. Assessment of map *a* using a comparison of the two half-maps and the Resmap program. Upper panel shows the local resolution using colouring as indicated by the key on the right. Resolution is worse on surface-exposed regions and better for the TM helical region. The density for ivacaftor is indicated in the TM region slice (right, dashed line). The resolution of the map in this region is around 5.5Å. The histogram at the bottom shows the number of voxels within the map that fall in regions associated with a specific spatial resolution. The lowest resolution bar in the histogram (far right) shows all voxels in the map from regions with resolution equal to or worse than 9Å.

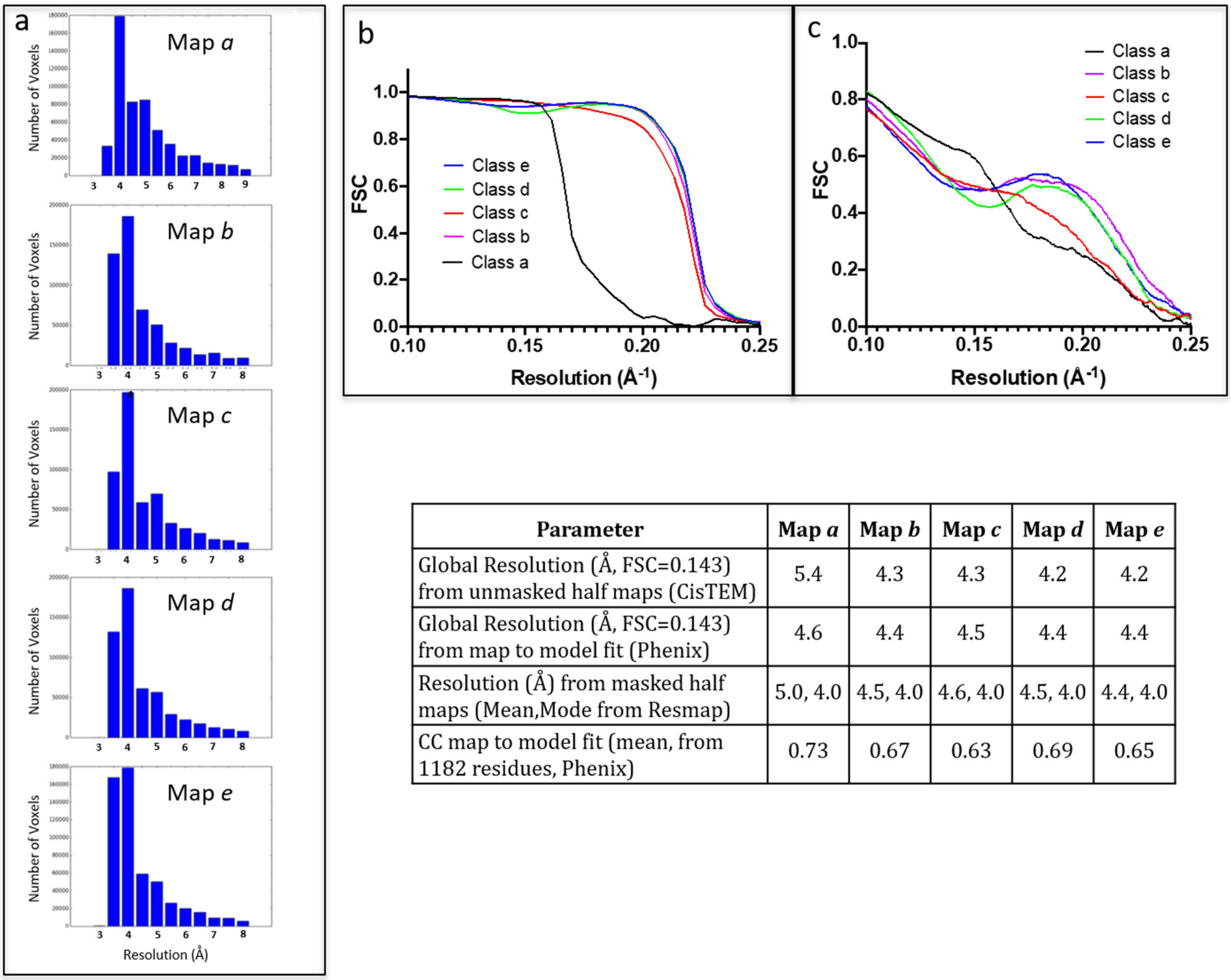

Parameter	Map *a*	Map *b*	Map *c*	Map *d*	Map *e*
Global Resolution (Å, FSC=0.143) from unmasked half maps (CisTEM)	5.4	4.3	4.3	4.2	4.2
Global Resolution (Å, FSC=0.143) from map to model fit (Phenix)	4.6	4.4	4.5	4.4	4.4
Resolution (Å) from masked half maps (Mean,Mode from Resmap)	5.0, 4.0	4.5, 4.0	4.6, 4.0	4.5, 4.0	4.4, 4.0
CC map to model fit (mean, from 1182 residues, Phenix)	0.73	0.67	0.63	0.69	0.65

Figure S7. Different assessments of the resolution of the five maps. **(a)** The histograms indicate the spread of resolution/variance across different regions of the maps using the Resmap program and comparing half-maps, each calculated wth half the particle numbers of the full maps. The number of voxels within 0.5Å resolution bands is plotted. The lowest resolution bar in each histogram (far right) shows all voxels from regions with resolution equal to or worse than the given resolution. **(b)** Resolution assessment using the Fourier shell correlation (FSC) calculated between the half-maps. **(c)** FSC plots for the relevant fitted model versus the full map. For reference, the table lower right reproduces Table I in the main text and summarises all the data for resolution assessment of the maps.

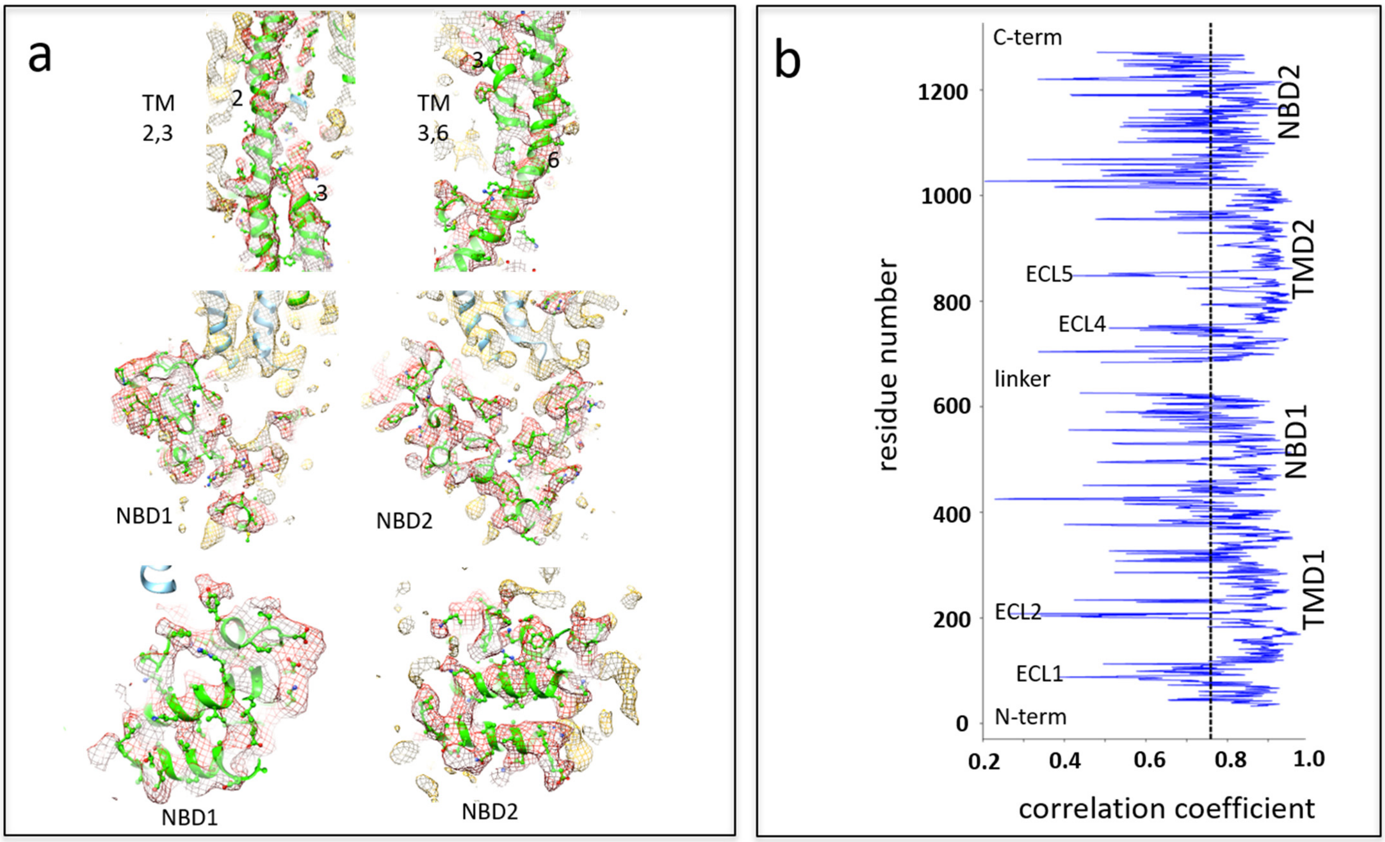

Figure S8. (a) Comparison of model-to-map fits for different regions of map *a*. Upper panels show transmembrane (TM) helices 2,3 and 6 (green ribbon trace depicts secondary structure, ball and stick representation of side-chain atoms) with the map (mesh) coloured red within 3Å of any model atom, yellow if not. Viewing direction is along the membrane plane. Middle panels show the NBD domain interfaces as viewed from between the NBDs. Colouring as previously but the intracellular loops formed by the cytoplasmic portions of TM helices are indicated with the blue ribbon. The lower panels show the helical sub-domain of the NBDs. **(b)** The panel shows the correlation coefficient (CC) calculated between individual residues and the local map density. The dashed line indicates the mean CC value. The TMD residues display slightly higher CC than the NBDs. Loops between secondary structural elements show the worst correlations, especially extracellular loops (ECL) 1,2,4 & 5.

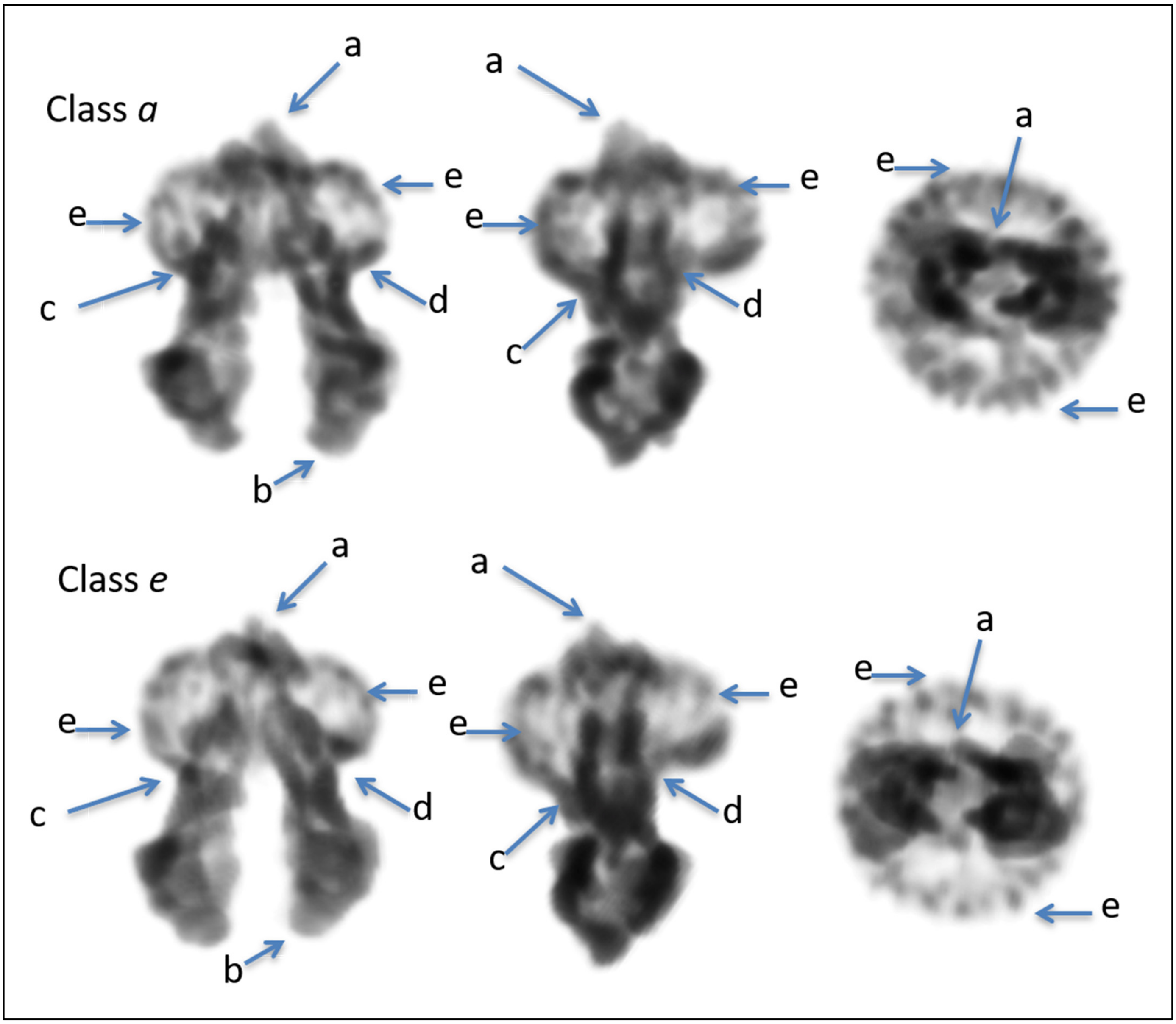

Figure S9. Asymmetry in P-gp. Examples of major asymmetrical features that distinguish between the N- and C-terminal halves of the protein and aid discrimination (by CisTEM) of alternative 180º-rotated projections of the protein/detergent complex (grey shading): **(a)** The large first extracellular loop. **(b)** The slightly narrower profile of NBD1 versus NBD2 (and elevated in class 5). **(c)** The C-terminal end of the NBD1-TMD2 linker and the elbow helix of TMD2. **(d)** Elbow helix and start of TM1. **(e)** Asymmetric micelle. Three different orthogonal projections are shown.
